# Synthesis of indole-based propellane derivatives via Weiss–Cook condensation, Fischer indole cyclization, and ring-closing metathesis as key steps

**DOI:** 10.3762/bjoc.9.307

**Published:** 2013-11-29

**Authors:** Sambasivarao Kotha, Ajay Kumar Chinnam, Arti Tiwari

**Affiliations:** 1 Department of Chemistry, Indian Institute of Technology-Bombay, Powai, India, Fax: 022-2572 7152

**Keywords:** allylation, indole derivatives, propellanes, ring-closing metathesis, Weiss–Cook condensation

## Abstract

A variety of highly functionalized indole-based [*n*.3.3]propellane derivatives is described. The synthesis of the propellane derivatives involves a Weiss–Cook condensation, a Fischer indole cyclization, and a ring-closing metathesis as key steps.

## Introduction

**Figure 1:**
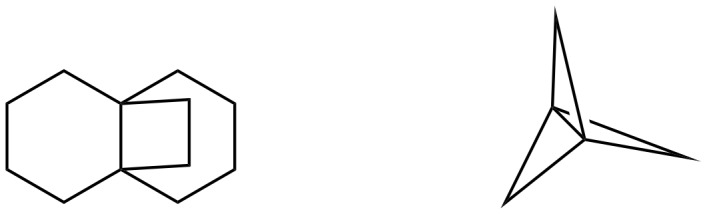
[4.4.2] and [1.1.1]propellanes.

**Figure 2:**
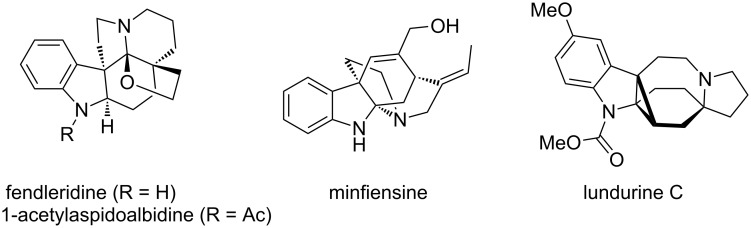
Alkaloids containing indole-based propellanes.

Propellanes are tricyclic systems conjoined with carbon–carbon single bonds (Figure 1) [1–3], and they are found to be highly congested. Some of these compounds are considered to be unstable entities and subjected to theoretical as well as synthetic studies [4]. Surprisingly, propellanes with larger rings, isolated from natural resources are found to be stable [5]. Among them, indole-based propellanes are useful in biology and medicine [6–12]. In 1963 Djerassi [13–14] isolated fendleridine and 1-acetylaspidoalbidine both of which belong to the aspidoalbine alkaloid family [15–20]. *Kopsia* alkaloids (Figure 2) show a wide range of structural diversity and exhibit interesting pharmacological activities [21]. For example, they are used for various ailments such as rheumatoid arthritis, edema, tonsillitis and hypertension. Lapidilectine B, grandilodine C [22], and lundurine B exhibit multidrug resistance (MDR) in vincristine-resistant KB cancer cells [23–24]. Minfiensine alkaloid [25–31] containing a novel 1,2,3,4-tetrahydrocarbazole ring skeleton shows anticancer activity [32]. König and co-workers identified some propellane derivatives as cannabinoid CB_1_ receptor antagonists [33], which are potential drugs for the treatment of schizophrenia and alcohol addiction [34]. However, the synthesis of indole alkaloid derivatives containing a propellane ring system is a challenging task due to the presence of quaternary centers involved with these systems [35].

**Figure 3:**
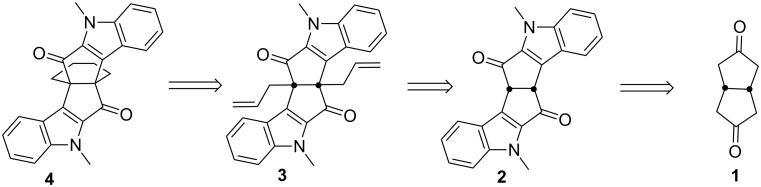
Retrosynthetic strategy to indole-based propellane **4**.

We envisioned a new synthetic route to diindole based propellane derivatives involving the Weiss–Cook condensation [36], Fischer indole cyclization [37–38], and ring-closing metathesis as key steps [39–42] (Figure 3).

Here, we report a new synthetic strategy to indole-based propellane derivatives and our approach has several points of diversification: (i) various aryl and heteroaryl fused indole derivatives can be assembled by choosing an appropriate hydrazine derivative, (ii) during the alkylation of diketone **2** [43] various unsaturated alkenyl fragments may be incorporated either in a symmetrical or in an unsymmetrical manner, (iii) various functionalized *cis*-bicyclo[3.3.0]octane-3,7-dione derivatives are available by the Weiss–Cook reaction, (iv) the double bond generated at the end of the RCM sequence provides an additional handle for further synthetic manipulation.

## Results and Discussion

**Scheme 1:**
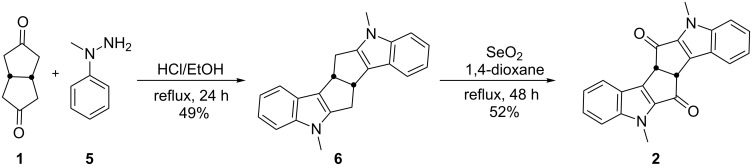
Preparation of diindole dione **2**.

To realize the strategy shown in Figure 3, *cis*-bicyclo[3.3.0]octane-3,7-dione (**1**) [44–50] was subjected to twofold Fischer indole cyclization to generate the diindole derivative **6** by using 1-methy-1-phenylhydrazine (**5**) under HCl/EtOH reflux conditions. Next, SeO_2_ oxidation of **6** in 1,4-dioxane under reflux gave the known diketone **2** (Scheme 1).

**Scheme 2:**
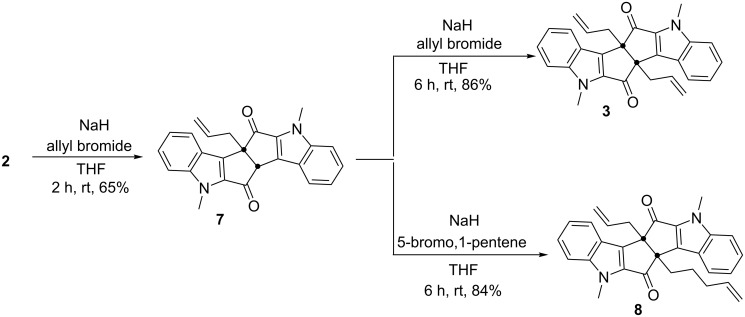
Synthesis of allylated indole derivatives **3**, **7** and **8**.

Later, diketone **2** was treated with allyl bromide in the presence of NaH to afford the mono-allylated product **7** in 65% yield. The allyl group attacks the molecule from the sterically less hindered convex side. Since the alkylation step can be performed stepwise, symmetrical as well as unsymmetrical diindole derivatives can be generated. Therefore, mono-allyl derivative **7** was subjected to a second allylation with allyl bromide to generate the diallyl diketone **3** (86%) (Scheme 2). The second allyl group is also placed from the convex side of the molecule. Later, the mono-allyl diketone **7** was treated with 5-bromo-1-pentene in the presence of NaH to generate the unsymmetrical diketone **8**.

**Scheme 3:**
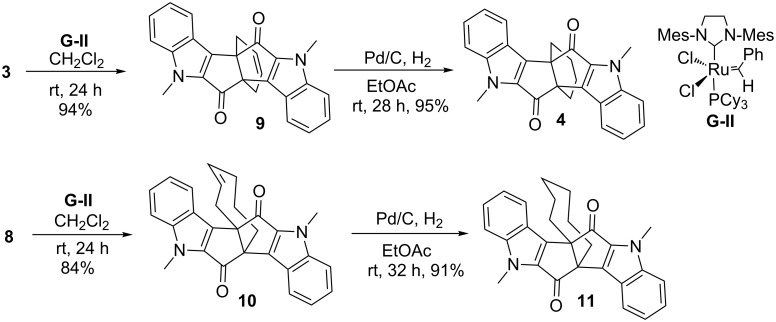
Synthesis of indole-based propellane derivatives **4** and **11** by RCM route.

Having compound **3** in hand, our next task was to construct the propellane derivatives via RCM by using Grubbs catalyst. In this regard, compound **3** was subjected to RCM under the influence of Grubbs 2^nd^ generation catalyst in dry CH_2_Cl_2_ to furnish the desired RCM product **9** in 94% yield. The unsaturated propellane derivative **9** was subjected to hydrogenation in the presence of 10% Pd/C in dry EtOAc under H_2_ atmosphere to afford the saturated propellane derivative **4** in 95% yield (Scheme 3). Along similar lines, propellane derivative **10** was synthesized from unsymmetrical diketone **8** by using Grubbs 2^nd^ generation catalyst and further subjected to hydrogenation to generate [6.3.3]propellane derivative **11**. It is noteworthy that the formation of the eight membered ring is generally considered a difficult task due to unfavourable entropy factors [51–53], but in our strategy the eight membered ring is successfully assembled with the aid of Grubbs 2^nd^ catalyst.

**Scheme 4:**
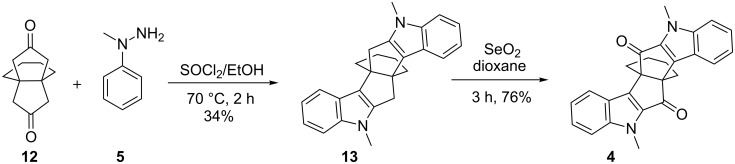
Synthesis of **4** by Weiss–Cook condensation and two fold Fischer indole cyclization.

Diindole derivative **4** was also synthesized by an independent route starting with [4.3.3]propellane derivative **12** obtained by Weiss–Cook condensation with cyclohexane-1,2-dione. Later, the propellane **12** was subjected to a twofold Fischer indole cyclization with 1-methyl-1-phenylhydrazine (**5**) with SOCl_2_/EtOH under reflux to deliver the diindole derivative **13** in 34% yield [54]. The diindole derivative **13** was subjected to SeO_2_ oxidation to generate the diketone **4** in 76% yield. The spectral data of the compound **4** obtained by this route (Scheme 4) is found to be identical with that of the compound obtained by the earlier route.

## Conclusion

We have developed a new and useful synthetic strategy to indole-based propellane derivatives with simple starting materials involving RCM as a key step. The structure of compound **4** has been elucidated based on spectral data and additionally by an independent synthetic sequence.

## Experimental

NMR spectra were recorded at rt on a 400 MHz Bruker NMR spectrometer in CDCl_3_ solution. Coupling constants (*J* values) are given in Hertz (Hz). Melting points were recorded with a Büchi melting point apparatus. Infrared (IR) spectra were recorded by using a Nicolet Impact-400 FTIR spectrometer in KBr. The high-resolution mass spectrometric measurements were carried out with a Micromass Q-ToF spectrometer. Analytical thin-layer chromatography (TLC) was performed on (10 × 5 cm) glass plates coated with Acme’s silica gel GF_254_ (containing 13% calcium sulfate as a binder). Chromatography was performed with Acme’s silica gel (100–200 mesh) by using double spray bellows for the application of pressure, and the column was eluted with an ethyl acetate/petroleum ether mixture. The organic solvents used in this study were dried over appropriate drying agents and distilled prior to use.

**6a-Allyl-*cis*-5,6a,11,12a-tetrahydro-5,11-dimethylpentaleno[2,1-*b*:5,4-*b*’]diindole-6,12-dione (7):** To a suspension of NaH (1.25 mmol) in THF (10 mL), diketone **2** (100 mg, 0.3 mmol) was added at room temperature under a nitrogen atmosphere. Then, the resulting reaction mixture was heated up to 65 °C for 15 min. After cooling to room temperature, allyl bromide (0.02 mL, 0.3 mmol) was added to the reaction mixture dropwise, and stirring was continued at room temperature for 2 h. At the end of the reaction (TLC monitoring), the reaction mixture was diluted with ethyl acetate (10 mL), washed with water and brine, dried over Na_2_SO_4_, and concentrated in vacuo. The crude product obtained was purified by silica gel column chromatography (5% EtOAc/petroleum ether) to give compound **7** (73 mg). Yellow colored solid; 65% yield. *R*_f_ = 0.42 (silica gel, 5% EtOAc/petroleum ether); mp: 176–178 °C; IR (KBr) ν*_max_*: 3023, 2986, 2934, 1735, 1447, 1248, 1048 cm^−1^; ^1^H NMR (400 MHz, CDCl_3_) δ 3.04–3.18 (m, 2H), 3.82 (s, 3H), 3.85 (s, 3H), 4.53 (s, 1H), 5.02 (dd, *J* = 10.0, 1.5 Hz, 1H), 5.19 (dd, *J* = 16.9, 1.4 Hz, 1H), 5.66–5.73 (m, 1H), 7.22–7.29 (m, 2H), 7.31–7.39 (m, 2H), 7.40–7.44 (m, 2H), 8.06–8.10 (m, 2H); ^13^C NMR (100 MHz, CDCl_3_) δ 30.39, 38.90, 58.73, 63.55, 111.18, 111.29, 119.24, 121.38, 121.40, 122.58, 123.09, 123.36, 123.51, 127.53, 127.64, 133.63, 134.92, 135.04, 138.90, 142.41, 144.94, 145.01, 189.86, 192.03; HRMS (ESI, Q-ToF) *m/z*: [M + H]^+^ calcd for C_25_H_21_N_2_O_2_, 381.1603; found, 381.1599.

**6a,12a-Diallyl-*cis*-5,6a,11,12a-tetrahydro-5,11-dimethylpentaleno[2,1-*b*:5,4-*b*’]diindole-6,12-dione (3):** To a suspension of NaH (1.25 mmol) in THF (10 mL), mono-allyl diketone **7** (70 mg, 0.18 mmol) was added at room temperature under a nitrogen atmosphere. Then, the resulting reaction mixture was heated up to 65 °C for 15 min. After cooling to room temperature, allyl bromide (0.02 mL, 0.27 mmol) was added to the reaction mixture in a dropwise manner, and stirring continued at room temperature for 6 h. At the end of the reaction (TLC monitoring), the reaction mixture was diluted with ethyl acetate (10 mL), washed with water and brine, dried over Na_2_SO_4_, and concentrated in vacuo. The crude product obtained was purified by silica gel column chromatography (5% EtOAc/petroleum ether) to deliver the compound **3** (67 mg). Yellow colored solid; 86% yield. *R*_f_ = 0.45 (silica gel, 5% EtOAc/petroleum ether); mp: 225–227 °C; IR (KBr) ν*_max_*: 2978, 2961, 2928, 1740, 1463, 1242, 1047 cm^−1^; ^1^H NMR (400 MHz, CDCl_3_) δ 3.17–3.23 (m, 2H), 3.44–3.49 (m, 2H), 3.82 (s, 6H), 4.98 (dd, *J* = 10.2, 1.2 Hz, 2H), 5.19 (dd, *J* = 17.1, 1.2 Hz, 2H), 5.65–5.72 (m, 2H), 7.20–7.29 (m, 2H), 7.32–7.37 (m, 2H), 7.39–7.41 (m, 2H), 8.04 (d, *J* = 8.0 Hz, 2H); ^13^C NMR (100 MHz, CDCl_3_) δ 30.28, 35.45, 67.02, 111.05, 117.76, 121.14, 122.51, 123.50, 127.26, 133.49, 134.54, 142.22, 144.61, 192.08.; HRMS (ESI, Q-ToF) *m/z*: [M + H]^+^calcd for C_28_H_25_N_2_O_2_, 421.1916; found, 421.1917.

**5,11-Dimethyl-6a,12a-but[2]enopentaleno[2,1-*b*:5,4-*b*']diindole-6,12(5*H*,11*H*)-dione (9):** A solution of diallyl dione **3** (65 mg, 0.15 mmol) in dry CH_2_Cl_2_ (15 mL) was degassed with N_2_ for 10 min, then, Grubbs 2^nd^ generation catalyst (12 mg, 0.014 mmol) was added at room temperature and stirred for 24 h. At the end of the reaction (TLC monitoring), the solvent was removed in vacuo and the crude product was purified by silica gel column chromatography (5% EtOAc/petroleum ether) to give compound **9** (57 mg). Colourless solid; 94% yield. *R*_f_ = 0.40 (silica gel, 5% EtOAc/petroleum ether); mp: 295–297 °C; IR (KBr) *ν_max_*: 3049, 2930, 1686, 1266, 1031 cm^−1^; ^1^H NMR (400 MHz, CDCl_3_) δ 2.86 (dd, *J* = 14.8, 2.6 Hz, 2H), 3.10 (dd, *J* = 14.2, 1.9 Hz, 2H), 3.83 (s, 6H), 5.95 (dd, *J* = 12.0, 8.8 Hz, 2H), 7.22–7.24 (m, 2H), 7.30–7.32 (m, 2H), 7.38–7.42 (m, 2H), 8.04 (m, 2H); ^13^C NMR (100 MHz, CDCl_3_) δ 30.41, 31.32, 64.23, 111.25, 121.26, 122.37, 123.37, 127.39, 128.25, 135.16, 142.35, 145.01, 192.57; HRMS (ESI, Q-ToF) *m/z*: [M + H]^+^ calcd for C_26_H_21_N_2_O_2_, 393.1603; found, 393.1609.

**5,11-Dimethyl-6a,12a-butanopentaleno[2,1-*b*:5,4-*b*']diindole-6,12(5*H*,11*H*)-dione (4):** To a solution of propellane **9** (50 mg, 0.12 mmol) in dry EtOAc (10 mL), 10% Pd/C (10 mg, 0.09 mmol) was added and the reaction mixture was stirred at room temperature under H_2_ atmosphere (1 atm) for 28 h. At the end of the reaction (TLC monitoring), the reaction mixture was filtered through a pad of celite and washed with ethyl acetate (20 mL). Evaporation of the solvent in vacuo gave the crude product. Further purification by silica-gel column chromatography (5% EtOAc/petroleum ether) gave the hydrogenated product **4** (48 mg). Colorless solid; 95% yield. *R*_f_ = 0.42 (silica gel, 5% EtOAc/petroleum ether); mp: 305–307 °C; IR (KBr) *ν_max_*: 2929, 2851, 1686, 1267, 1047 cm^−1^; ^1^H NMR (400 MHz, CDCl_3_) δ 1.49–1.54 (m, 2H), 1.58–1.63 (m, 2H), 2.26–2.34 (m, 2H), 2.50–2.57 (m, 2H), 3.84 (s, 6H), 7.21–7.27 (m, 2H), 7.31–7.34 (m, 2H), 7.38–7.42 (m, 2H), 8.06 (d, *J* = 8.1 Hz, 2H); ^13^C NMR (100 MHz, CDCl_3_) δ 17.16, 28.07, 30.25, 63.11, 111.04, 121.00, 122.56, 123.30, 127.19, 134.76, 142.73, 144.80, 193.29; HRMS (ESI, Q-ToF) *m/z*: [M + H]^+^ calcd for C_26_H_23_N_2_O_2_, 395.1760; found, 395.1750.

To a solution of propellane **13** (33 mg, 0.09 mmol) in dioxane (10 mL), SeO_2_ (22 mg, 0.20 mmol) was added and the reaction mixture was refluxed for 3 h. At the end of the reaction (TLC monitoring), the reaction mixture was filtered through a pad of celite and washed with a 1:1 mixture of CCl_4_ and CHCl_3_ (20 mL). Evaporation of the solvent in vacuo gave the crude product, which was further purified by silica-gel column chromatography. Elution of the column with 5% EtOAc/petroleum ether gave the diketone **4** (27 mg, 76%) as a white solid. The spectral data of this compound is identical with that of compound **4** obtained by the other route.

## Supporting Information

File 1 Copies of ^1^H, ^13^C NMR and HRMS spectra for all new compounds.
