# Supplementary material for: Synthesis of indole-based propellane derivatives via Weiss–Cook condensation, Fischer indole cyclization, and ring-closing metathesis as key steps
Source: Beilstein J Org Chem. 2013 Nov 29;9:2709–14. doi: 10.3762/bjoc.9.307 (PMC3869257; doi:10.3762/bjoc.9.307)
Supplement: File 1 — Copies of 1H, 13C NMR and HRMS spectra for all new compounds. [file Beilstein_J_Org_Chem-09-2709-s001.pdf]

Supporting Information

for

Synthesis of indole-based propellane derivatives via Weiss–Cook condensation, Fischer indole cyclization, and ring-closing metathesis as key steps

Sambasivarao Kotha*, Ajay Kumar Chinnam and Arti Tiwari

Address: Department of Chemistry, Indian Institute of Technology-Bombay, Powai, India, Fax: 022-2572 7152

Email: Sambasivarao Kotha - srk@chem.iitb.ac.in

*Corresponding author

Copies of ^{1}H, ^{13}C NMR and HRMS spectra for all new compounds

Table of contents

1. Synthetic scheme and Experimental data for compound 11 S2–S4
2. ^{1}H, ^{13}C NMR and HRMS of compound **7** S4–S5
3. ^{1}H, ^{13}C NMR and HRMS of compound **3** S6–S7
4. ^{1}H, ^{13}C NMR and HRMS of compound **9** S8–S9
5. ^{1}H, ^{13}C NMR and HRMS of compound **4** S10–S11
6. ^{1}H, ^{13}C NMR and HRMS of compound **8** S12–S13
7. ^{1}H, ^{13}C NMR and HRMS of compound **10** S14–S15
8. ^{1}H, ^{13}C NMR and HRMS of compound **11** S16–S17

1. Synthetic scheme and experimental data for compound 11

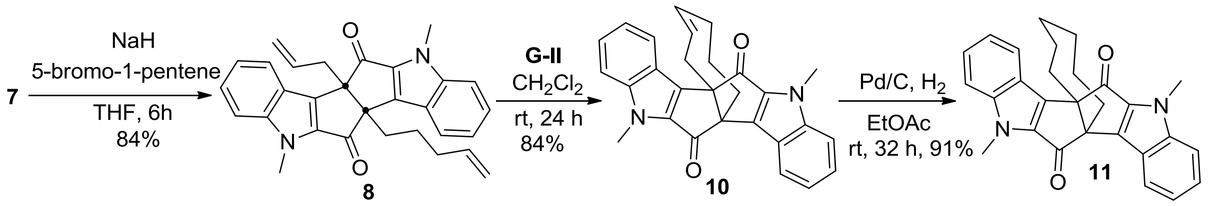

6a-Allyl-12a-(pent-4-en-1-yl)-*cis*-5,6a,11,12a-tetrahydro-5,11-dimethylpentaleno[2,1-*b*:5,4-*b*']diindole-6,12-dione (8)

To a suspension of NaH (1.25 mmol) in THF (10 mL), mono-allyl dione **7** (100 mg, 0.26 mmol) was added at room temperature under nitrogen atmosphere. Then, the resulting reaction mixture was heated up to 65 °C for 15 min. After cooling to room temperature, 5-bromo-1-pentene (0.04 mL, 0.39 mmol) was added to the reaction mixture dropwise, and the stirring was continued at room temperature for 6 h. At the end of the reaction (TLC monitoring), the reaction mixture was diluted with ethyl acetate (10 mL), washed with water and brine, dried over Na_2SO_4 and concentrated in vacuo. The crude product obtained was purified by silica gel column chromatography (5% EtOAc/petroleum ether) to give compound **8** (77 mg).

Yellow colour solid; 84% yield.

R_f = 0.41(silica gel, 5% EtOAc/petroleum ether); mp:161-163 ^oC.

IR (KBr) ν_{max}: 3056, 2927, 2857, 1686, 1459, 1216 cm^{-1}.

^1H NMR (400 MHz, $CDCl_3$): δ 1.38-1.55 (2H, m), 2.05-2.10 (2H, m), 2.33-2.40 (1H, m), 2.58-2.69 (1H, m), 3.09-3.15 (1H, m), 3.46-3.52 (1H, m), 3.82 (3H, s), 3.83 (3H, s), 4.91-5.00 (3H, m), 5.19 (1H, dd, J = 17.0, 1.4 Hz), 5.63-5.78 (2H, m), 7.19-7.24 (2H, m), 7.28-7.31 (2H, m), 7.36-7.41 (2H, m), 8.01-8.04 (2H, m).

^{13}C NMR (100 MHz, $CDCl_3$): δ 25.72, 30.42, 31.02, 34.53, 36.44, 66.99, 67.98, 111.20, 115.25, 117.83, 121.26, 121.30, 122.71, 122.96, 123.62, 127.33, 127.39, 134.80, 138.40, 142.29, 143.03, 144.70, 192.39, 192.98.

HRMS (ESI, Q-ToF): *m/z* calcd for $C_{30}H_{29}N_2O_2$ $[M+H]^+$ 449.2229 found: 449.2224.

Synthesis of compound 10

A solution of diketone **8** (70 mg, 0.15 mmol) in dry CH_2Cl_2 (15 mL) was degassed with N_2 for 10 min, then Grubbs 2nd generation catalyst (11 mg, 0.012 mmol) was added at room temperature and the reaction mixture was stirred for 24 h. At the end of the reaction (TLC monitoring), the solvent was removed in vacuo and the crude product was purified by silica gel column chromatography (5% EtOAc/petroleum ether) to give the RCM product (55 mg).

Colourless solid; 84% yield.

R_f = 0.39 (silica gel, 5% EtOAc-petroleum ether); mp: 227-229 ^oC.

IR (KBr) ν_{max}: 3054, 2988, 2929, 1685, 1266, 1020 cm^{-1}.

^1H NMR (400 MHz, $CDCl_3$): δ 1.74-1.81 (2H, b,m), 2.10-2.15 (2H, m), 2.46-2.53 (1H, m), 2.78-2.91 (1H, m), 3.10-3.23 (2H, m), 3.81 (6H, s), 5.47 (1H, d, J = 6.7 Hz), 5.64-5.70 (1H, m), 7.19-7.27 (2H, m), 7.29-7.31 (2H, m), 7.36-7.41 (2H, m), 8.01 (1H, d, J = 7.9 Hz), 8.09 (1H, d, 8.0 Hz).

^{13}C NMR (100 MHz, $CDCl_3$): δ 26.46, 26.61, 27.99, 28.16, 28.85, 29.87, 30.29, 30.38, 30.40, 35.59, 35.96, 69.42, 70.93, 111.21, 121.16, 121.26, 122.44, 123.00, 123.33, 123.40, 127.23, 127.84, 133.38, 134.01, 142.37, 144.65, 144.72, 192.57, 192.90.

HRMS (ESI, Q-ToF): m/z calcd for $C_{28}H_{25}N_2O_2$ $[M+H]^+$ 421.1908 found: 421.1908.

Synthesis of compound 11

To a solution of propellane **10** (45 mg, 0.10 mmol) in dry EtOAc (10 mL), 10% Pd/C (9 mg, 0.08 mmol) was added and the reaction mixture was stirred at room temperature under H_2 atmosphere (1 atm) for 32 h. At the end of the reaction (TLC monitoring), the reaction mixture was filtered through a pad of celite and washed with ethyl acetate (20 mL). Evaporation of the solvent in vacuo gave the crude product, which was further purified by silica-gel column chromatography (5% EtOAc/petroleum ether) to give the hydrogenated product **11** (41 mg).

Colourless solid; 91% yield.

R_f = 0.41 (silica gel, 5% EtOAc/petroleum ether); mp>339 ^oC decomposing.

IR (KBr) ν_{max}: 3054, 2929, 1685, 1266, 1020 cm^{-1}.

^{1}H NMR (400 MHz, $CDCl_3$): δ 1.24-1.31 (6H, m), 1.82-1.86 (2H, m), 2.61 (4H, t, J = 5.9 Hz), 3.81 (6H, s), 7.20-7.26 (2H, m), 7.29-7.31 (2H, m), 7.36-7.40 (2H, m), 8.06 (2H, d, J=8.1 Hz).

^{13}C NMR (100 MHz, $CDCl_3$): δ 26.28, 27.19, 30.09, 30.38, 67.89, 111.25, 121.22, 122.88, 123.47, 127.18, 133.45, 143.35, 144.73, 193.35.

HRMS (ESI, Q-ToF): *m/z* calcd for $C_{28}H_{25}N_2O_2$ $[M+H]^+$423.2073 found: 423.2071.

2. ^{1}H, ^{13}C and HRMS of compound 7

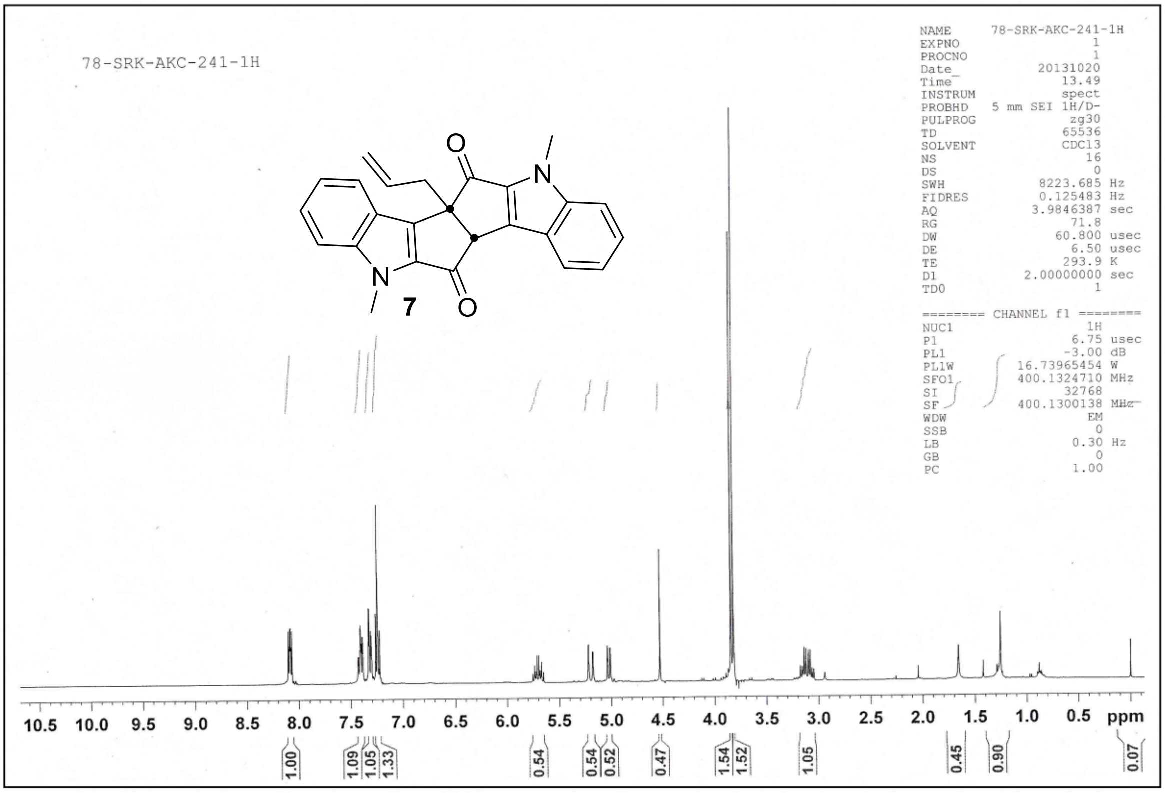

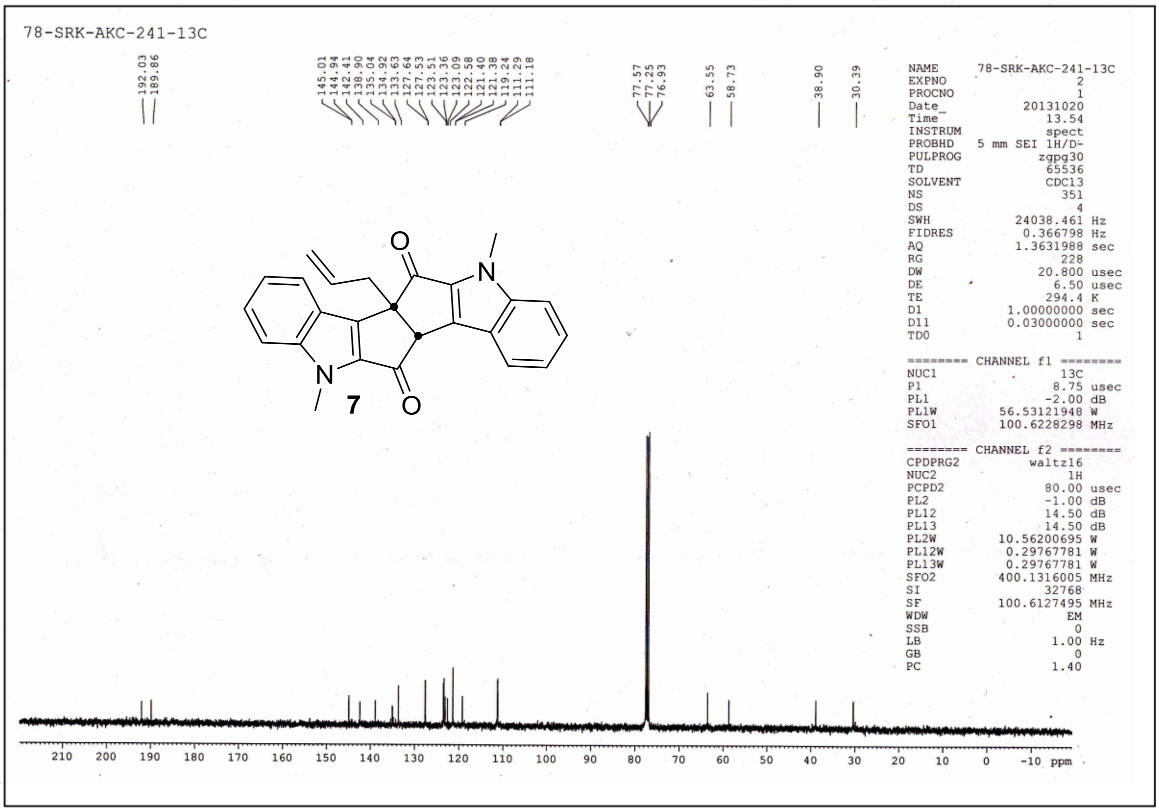

Elemental Composition Report **Page 1**

Single Mass Analysis (displaying only valid results)
Tolerance = 30.0 PPM / DBE: min = -1.5, max = 200.0
Isotope cluster parameters: Separation = 1.0 Abundance = 1.0%

Monoisotopic Mass, Odd and Even Electron Ions
20 formula(e) evaluated with 1 results within limits (all results (up to 1000) for each mass)

Waters(Micromass) : Q-Tof micro(YA-105) Dept. Of Chemistry - I.I.T.(B) 03-Sep-201215:39:37
C25H20N2O2
SRK-AKC-68-51 32 (0.317) AM (Cen,5, 80.00, Ht,5000.0,556.28,1.00); Sm (Mn, 2x4.00); Sb (5,40.00); Cm (1:34) TOF MS ES+ 812

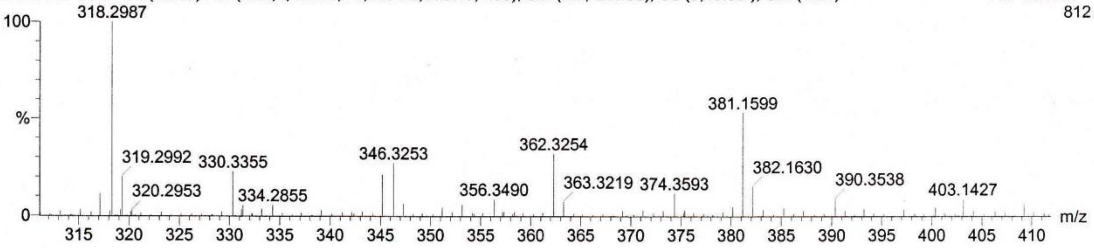

Minimum:				-1.5		
Maximum:		200.0	30.0	200.0		
Mass	Calc. Mass	mDa	PPM	DBE	Score	Formula
381.1599	381.1603	-0.4	-1.0	16.5	1	C25 H21 N2 O2

3. ^{1}H, ^{13}C and HRMS of compound 3

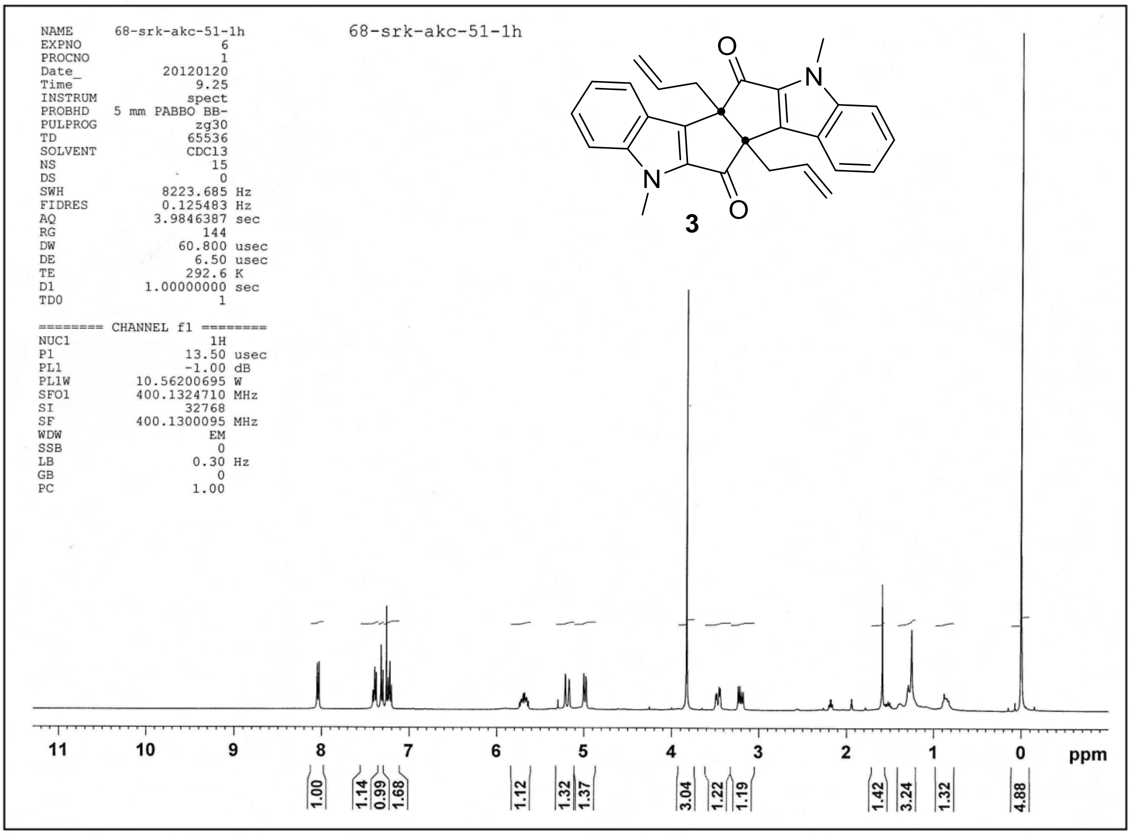

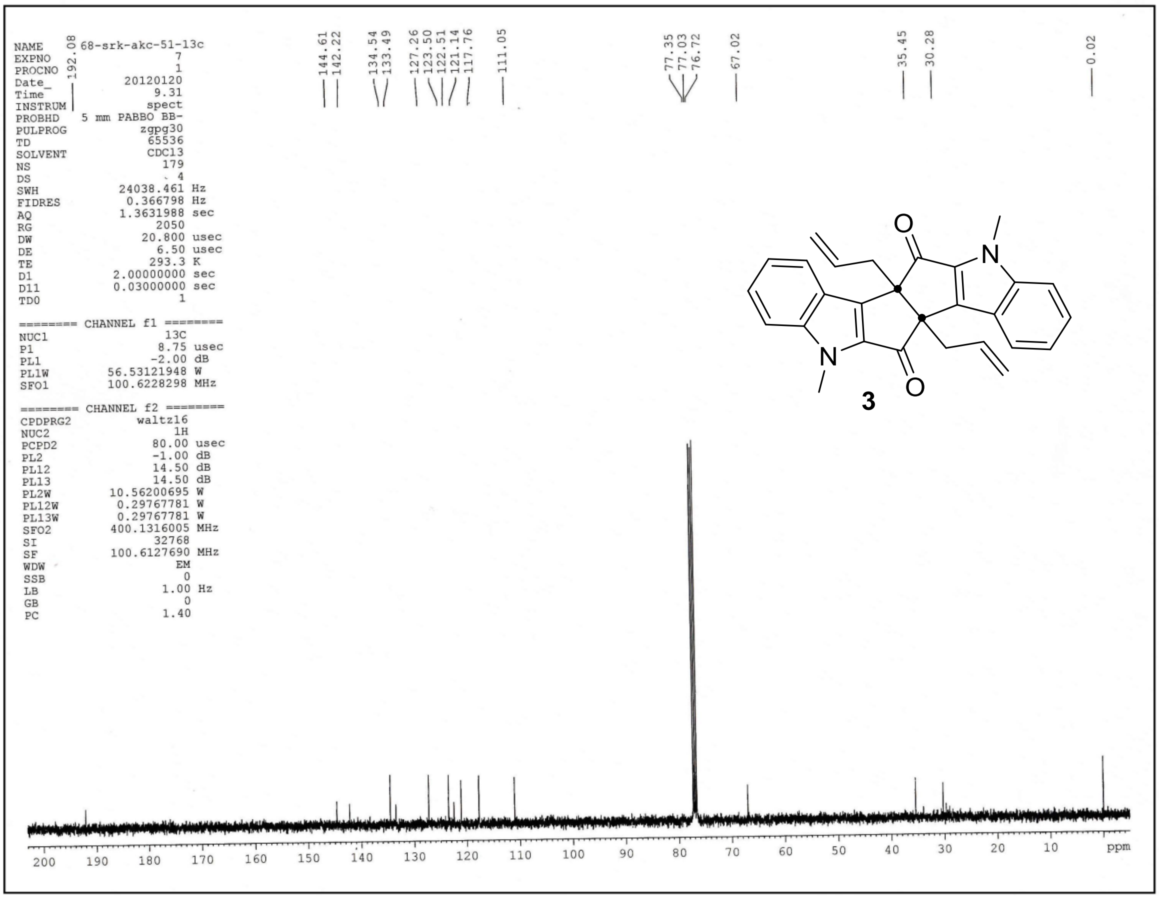

Elemental Composition Report

Page 1

Single Mass Analysis (displaying only valid results)

Tolerance = 5.0 PPM / DBE: min = -1.5, max = 200.0

Isotope cluster parameters: Separation = 1.0 Abundance = 1.0%

Monoisotopic Mass, Odd and Even Electron Ions

594 formula(e) evaluated with 1 results within limits (all results (up to 1000) for each mass)

Waters(Micromass) : Q-Tof micro(YA-105) Dept. Of Chemistry - I.I.T.(B) 24-Sep-201215:25:37

C28H24N2O2

SRK-AKC-68-56 37 (0.371) AM (Cen,6, 80.00, Ht,5000.0,556.28,1.00); Sb (3,40.00); Cm (1:38)

TOF MS ES+

3.97e3

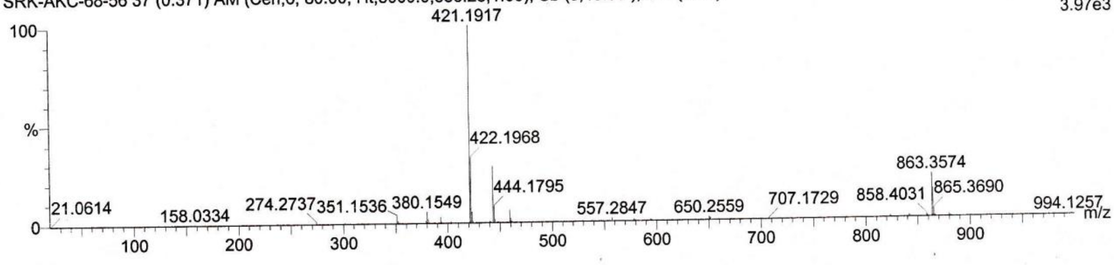

Minimum:				-1.5		
Maximum:		200.0	5.0	200.0		
Mass	Calc. Mass	mDa	PPM	DBE	Score	Formula
421.1917	421.1916	0.1	0.3	17.5	1	C28 H25 N2 O2

4. ^{1}H, ^{13}C and HRMS of compound 9

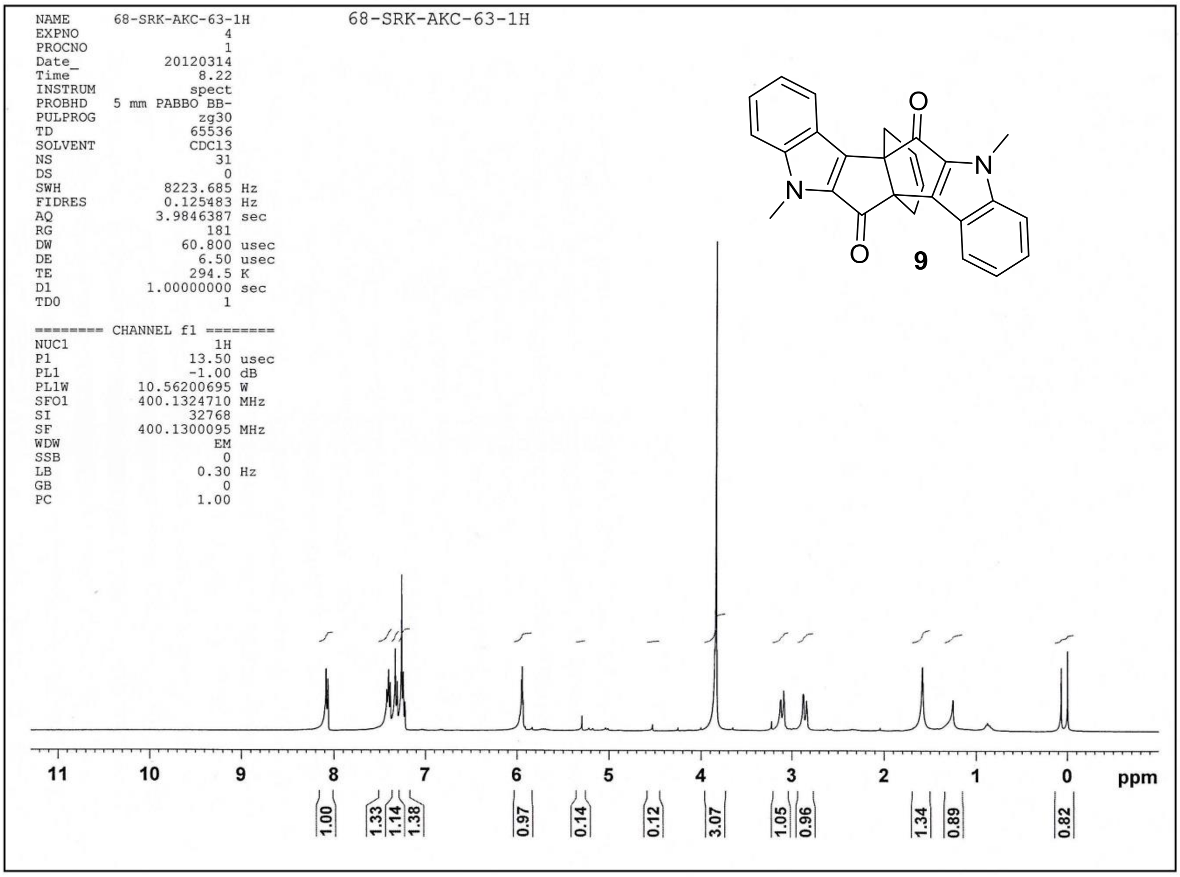

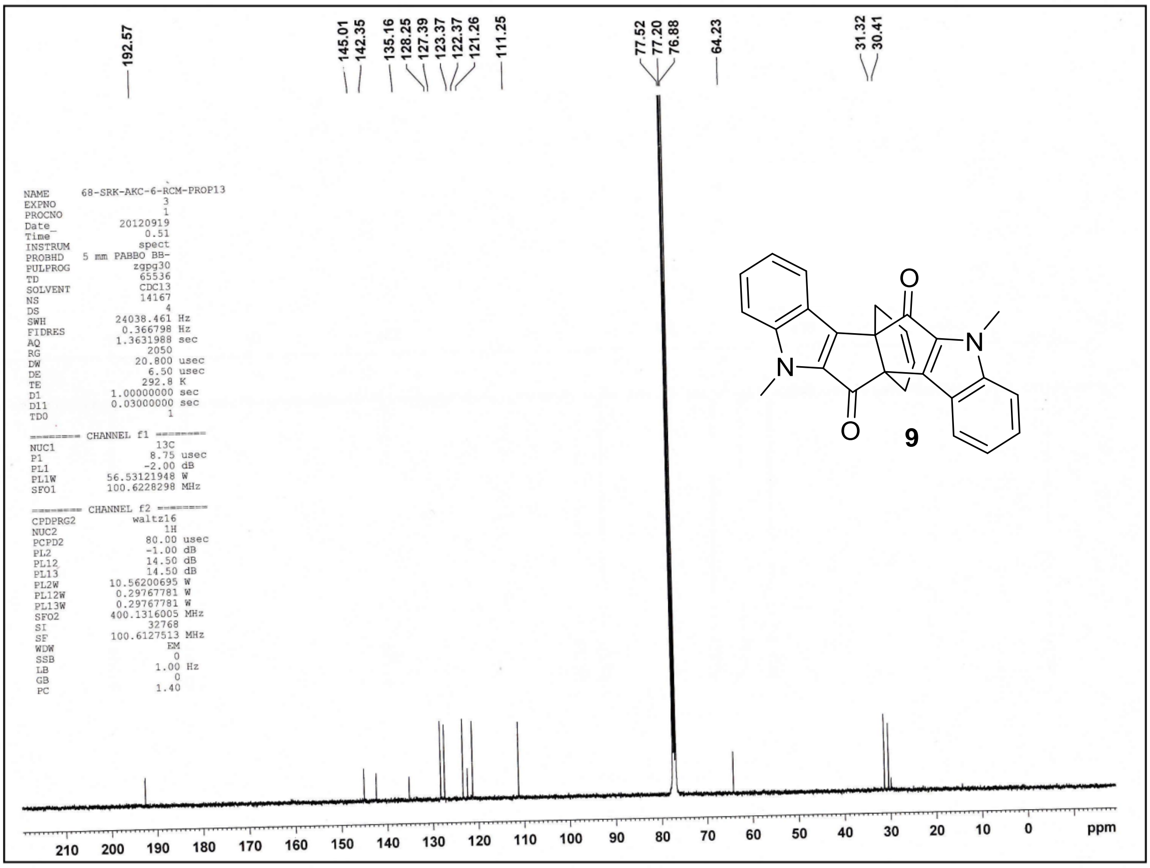

3.3. Rcm

Elemental Composition Report

Page 1

Single Mass Analysis (displaying only valid results)

Tolerance = 10.0 PPM / DBE: min = -1.5, max = 200.0
Isotope cluster parameters: Separation = 1.0 Abundance = 1.0%

Monoisotopic Mass, Odd and Even Electron Ions
4 formula(e) evaluated with 1 results within limits (up to 50 closest results for each mass)

Micromass : Q-Tof micro (YA-105) Dept. Of Chemistry I.I.T.(B)
C26H20N2O2 20-Apr-201210:06:17
SRK-AKC-68-63 5 (0.049) AM (Cen,5, 80.00, Ht,5000.0,556.28,1.00); Sb (5,40.00); Cm (1:30) TOF MS ES+ 4.34e3

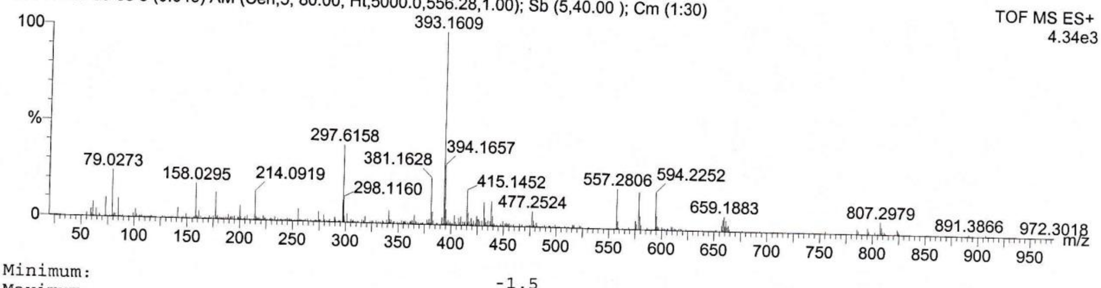

Minimum:				-1.5		
Maximum:		200.0	10.0	200.0		
Mass	Calc. Mass	mDa	PPM	DBE	Score	Formula
393.1609	393.1603	0.6	1.6	17.5	1	C26 H21 N2 O2

5. ^{1}H, ^{13}C and HRMS of compound 4

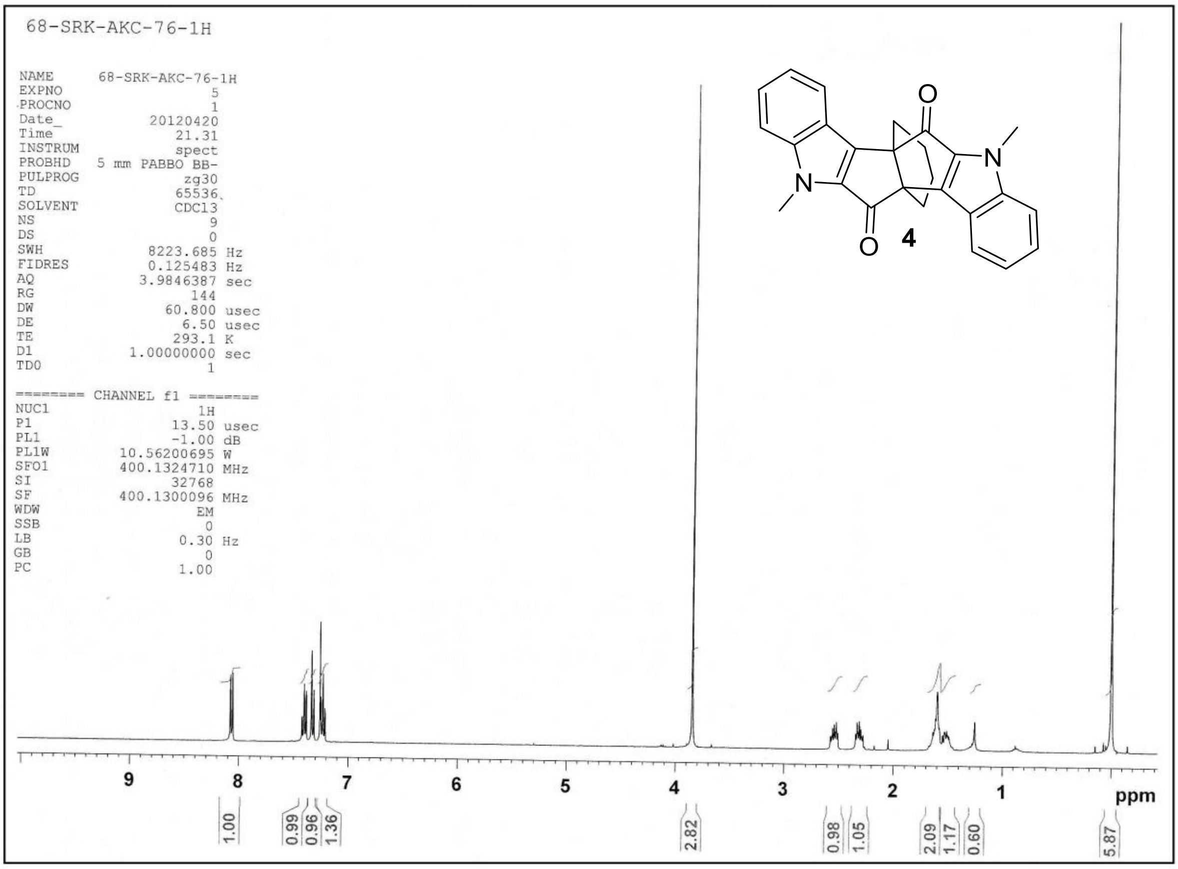

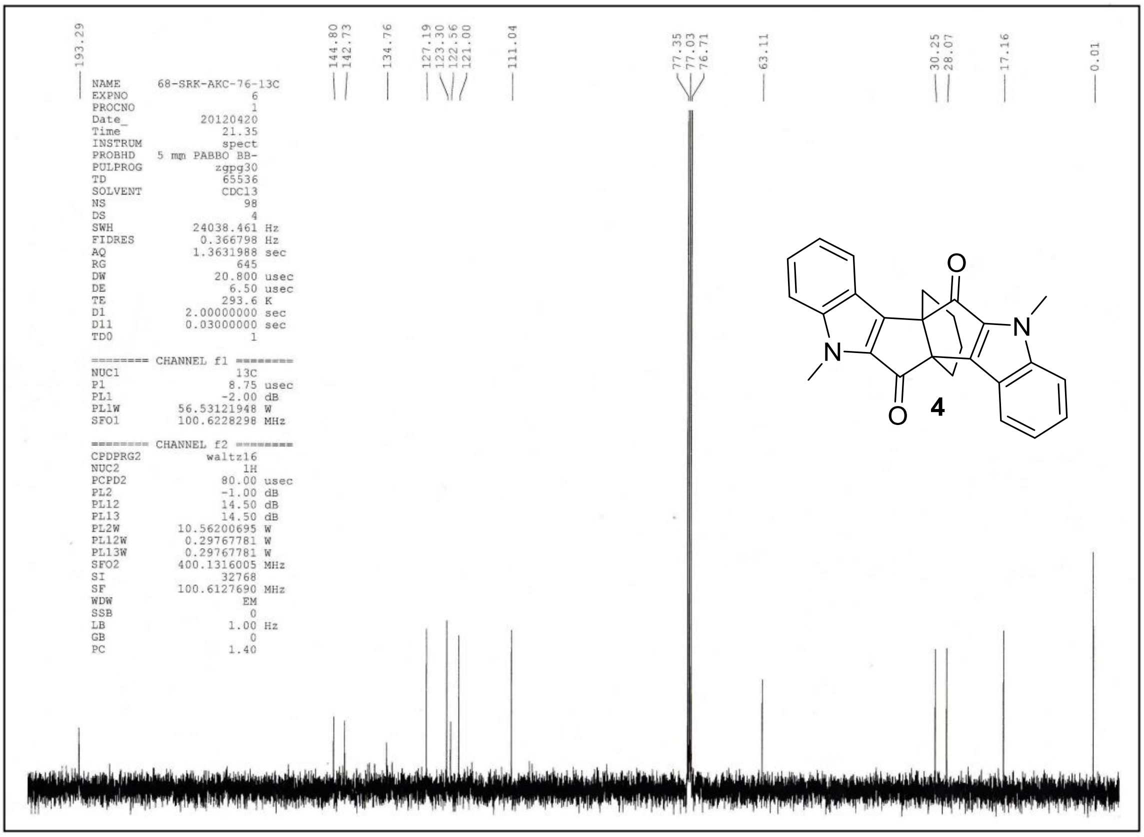

Elemental Composition Report

Page 1

Single Mass Analysis (displaying only valid results)

Tolerance = 5.0 PPM / DBE: min = -1.5, max = 200.0
Isotope cluster parameters: Separation = 1.0 Abundance = 1.0%

Monoisotopic Mass, Odd and Even Electron Ions
22 formula(e) evaluated with 1 results within limits (all results (up to 1000) for each mass)

Waters(Micromass) : Q-Tof micro(YA-105) Dept. Of Chemistry - I.I.T.(B) 03-Sep-201212:31:22
C26H22N2O2
SRK-AKC-68-66 31 (0.306) AM (Cen,5, 80.00, Ar,5000.0,556.28,1.00); Sm (Mn, 2x4.00); Sb (5,40.00); Cm (1:34) TOF MS ES+ 2.52e4

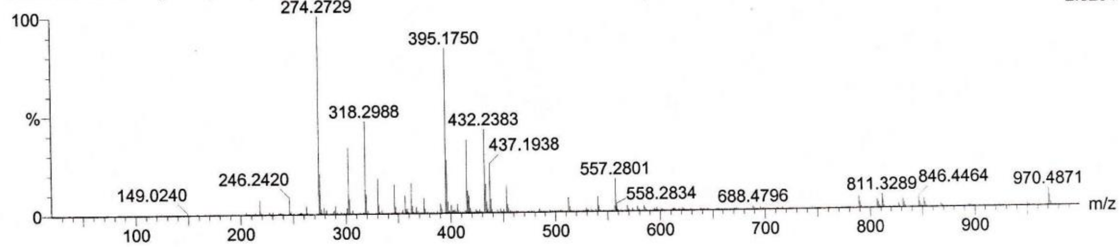

Minimum:				-1.5		
Maximum:		200.0	5.0	200.0		
Mass	Calc. Mass	mDa	PPM	DBE	Score	Formula
395.1750	395.1760	-1.0	-2.4	16.5	1	C26 H23 N2 O2

6. ^{1}H, ^{13}C and HRMS of compound 8

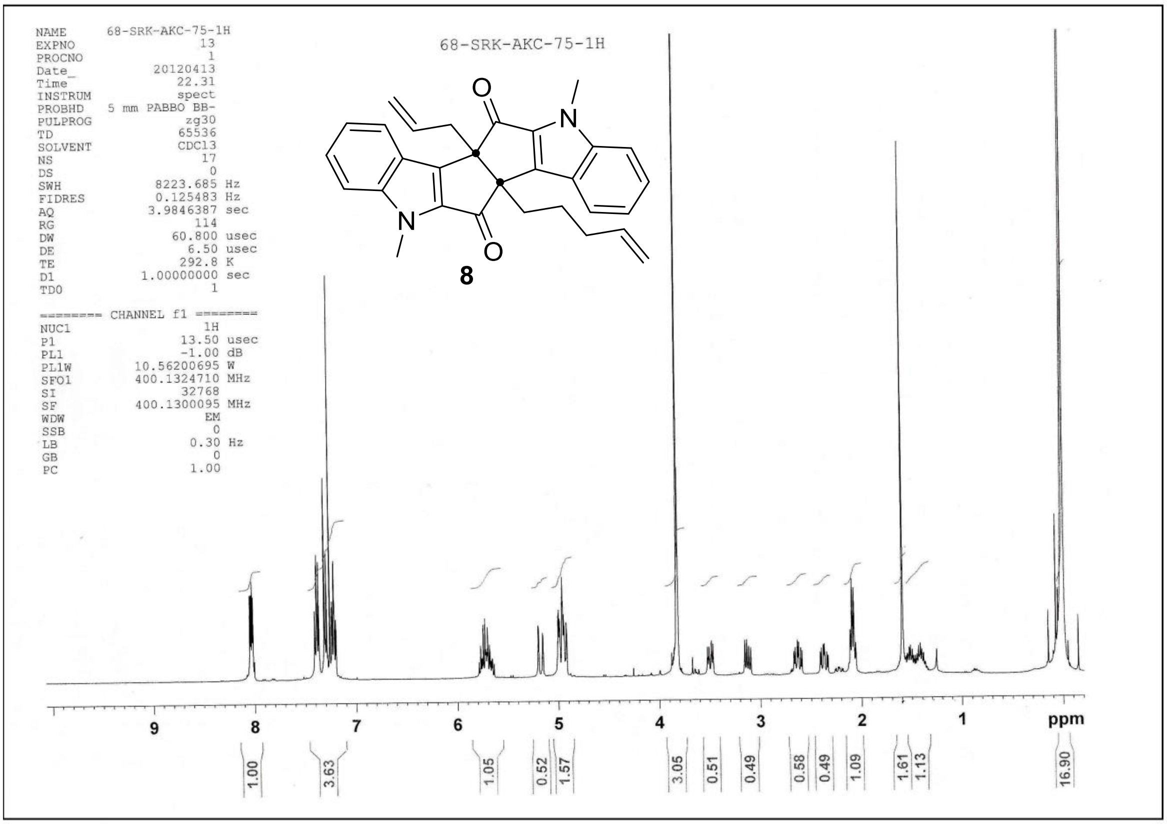

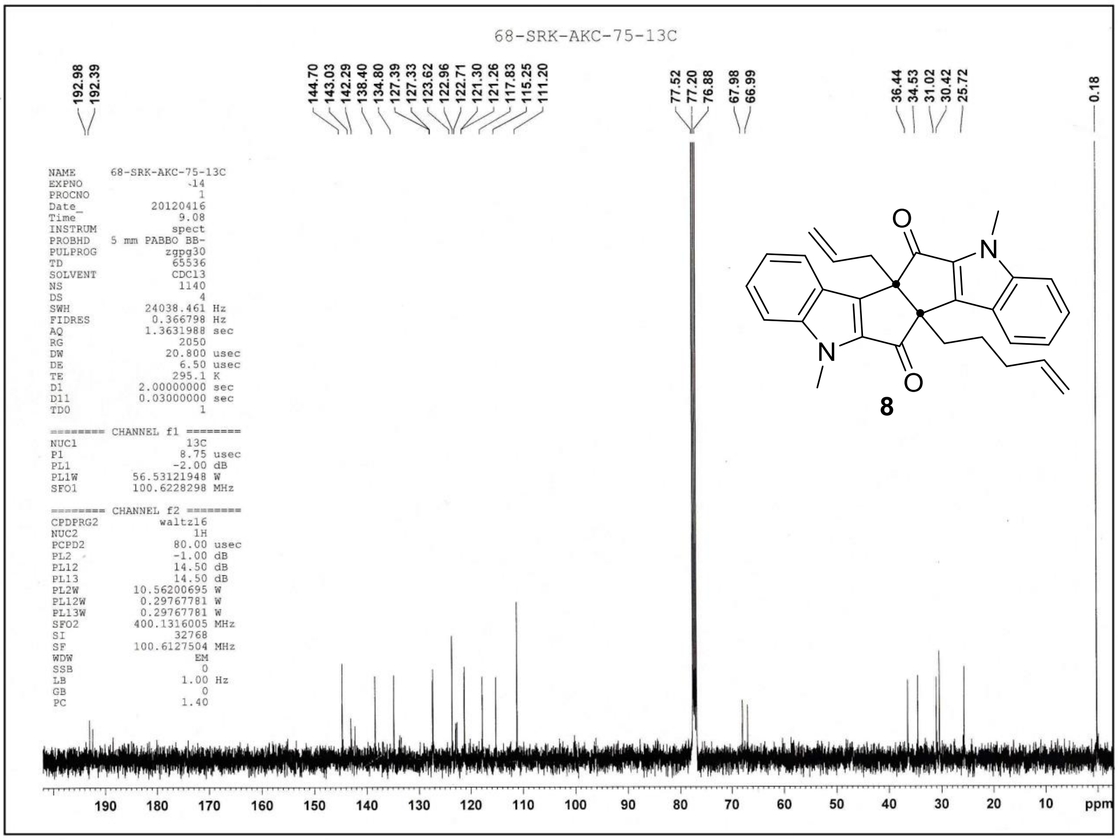

Elemental Composition Report **Page 1**

Single Mass Analysis (displaying only valid results)

Tolerance = 50.0 PPM / DBE: min = -1.5, max = 200.0

Isotope cluster parameters: Separation = 1.0 Abundance = 1.0%

Monoisotopic Mass, Odd and Even Electron Ions

4 formula(e) evaluated with 1 results within limits (up to 50 closest results for each mass)

Micromass : Q-Tof micro (YA-105) Dept. Of Chemistry I.I.T.(B) 10-May-201211:30:34

C30H29N2O2

SRK-AKC-68-73 47 (0.469) AM (Top,5, Ht,5000.0,556.28,1.00); Sm (Md, 6.00); Sb (5,40.00) TOF MS ES+ 92

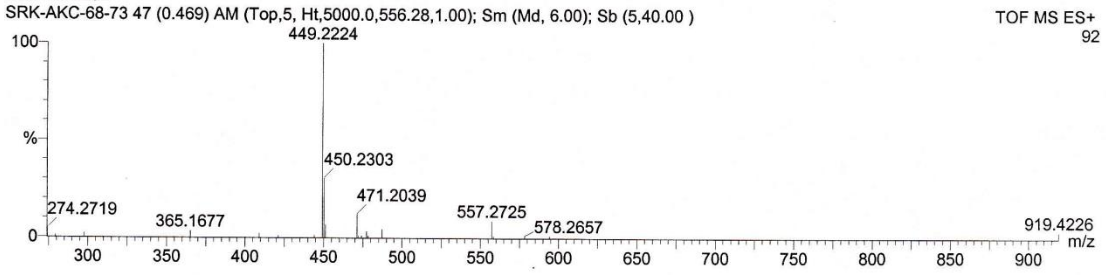

Minimum:				-1.5		
Maximum:		200.0	50.0	200.0		
Mass	Calc. Mass	mDa	PPM	DBE	Score	Formula
449.2224	449.2229	-0.5	-1.1	17.5	1	C30 H29 N2 O2

7. ^{1}H, ^{13}C and HRMS of compound 10

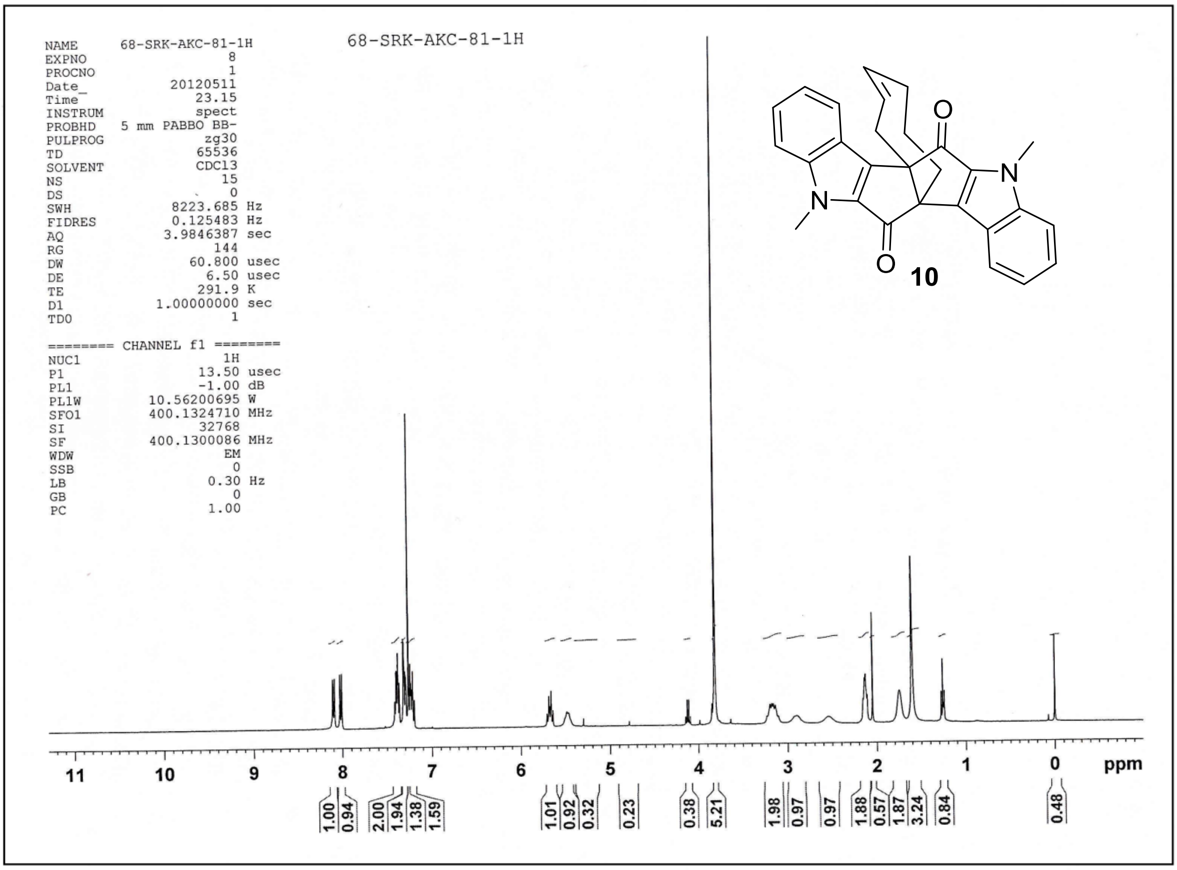

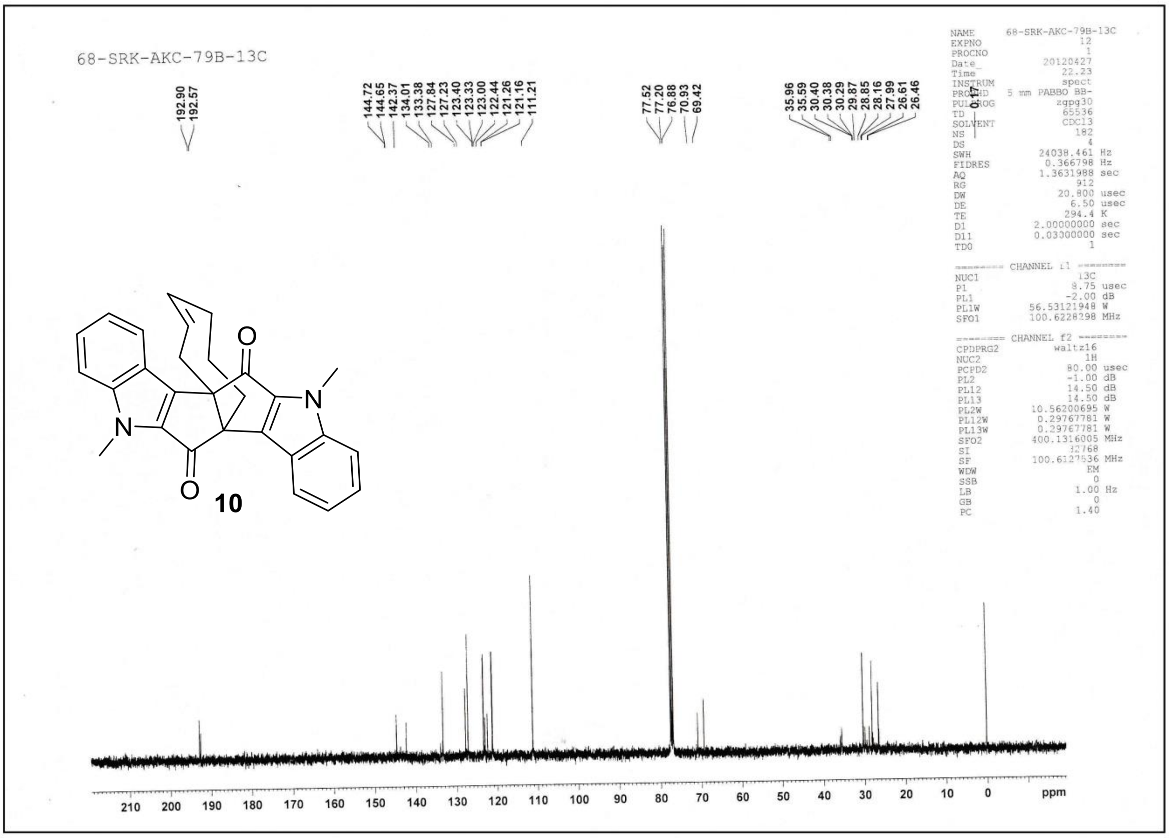

calculated mass(421.1908)

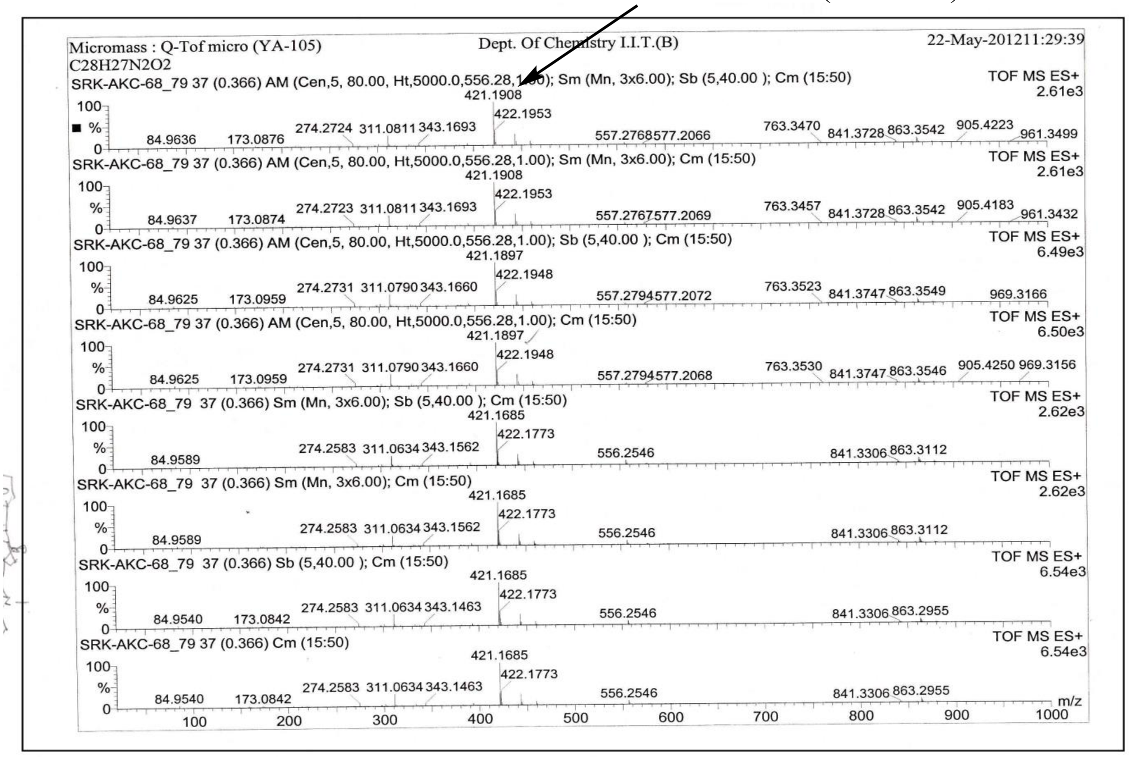

8. ^{1}H, ^{13}C and HRMS of compound 11

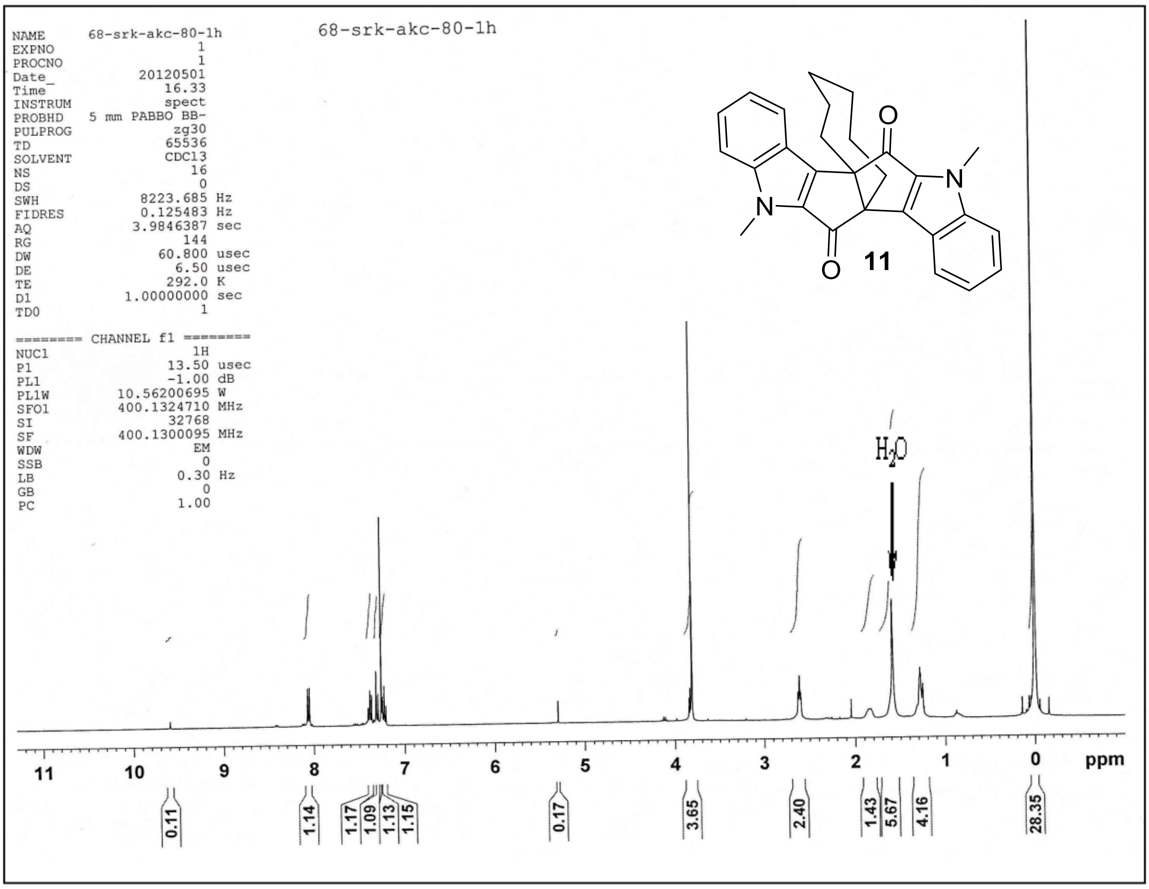

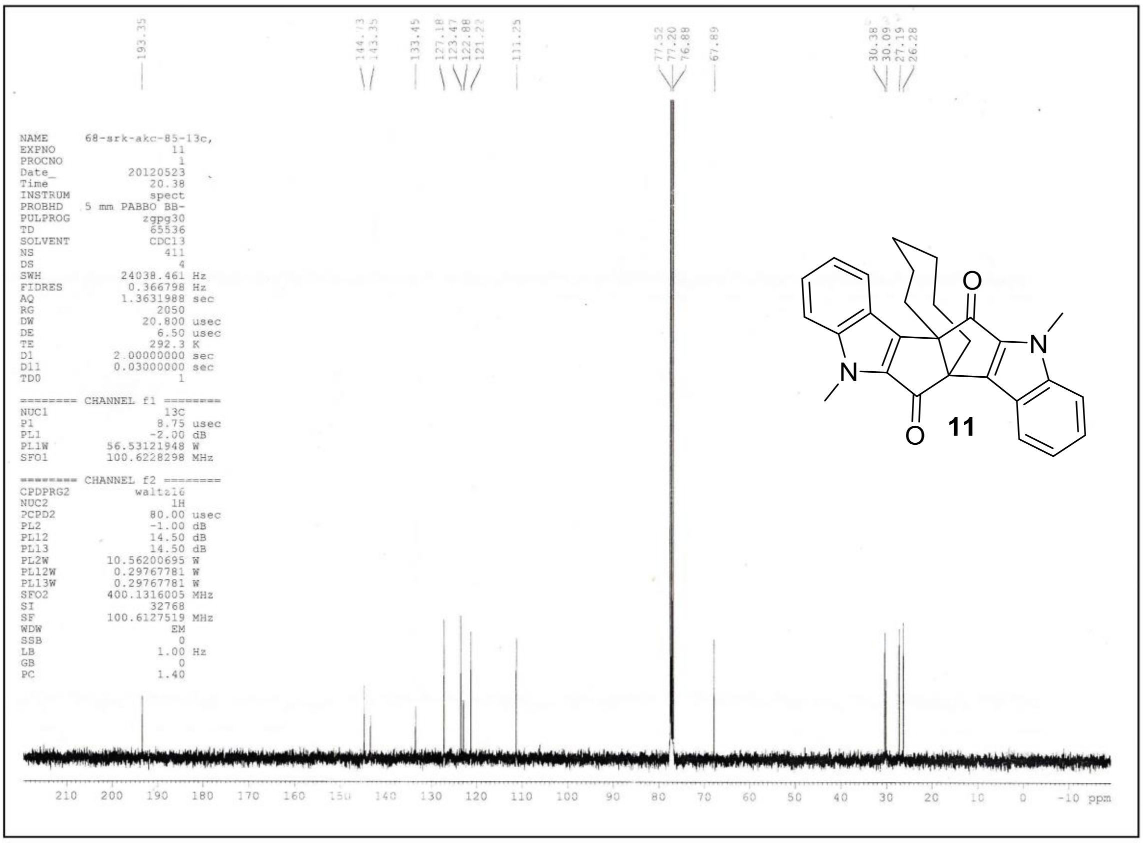

Elemental Composition Report

Page 1

Single Mass Analysis (displaying only valid results)

Tolerance = 50.0 PPM / DBE: min = -1.5, max = 200.0

Isotope cluster parameters: Separation = 1.0 Abundance = 1.0%

Monoisotopic Mass, Odd and Even Electron Ions

4 formula(e) evaluated with 1 results within limits (up to 50 closest results for each mass)

Micromass : Q-Tof micro (YA-105) Dept. Of Chemistry I.I.T.(B) 22-May-201211:10:54

C28H29N2O2

SRK-AKC-68-80 21 (0.208) AM (Cen,5, 80.00, Ht,5000.0,556.28,1.00); Sm (Mn, 3x6.00); Sb (5,40.00); Cm (1:31) TOF MS ES+ 2.07e3

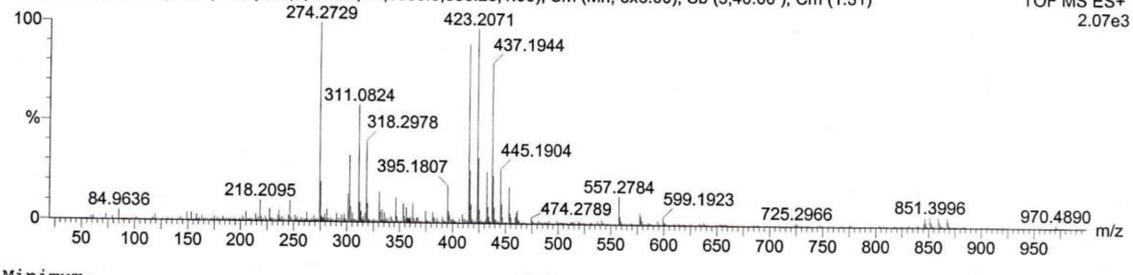

Minimum:				-1.5		
Maximum:		200.0	50.0	200.0		
Mass	Calc. Mass	mDa	PPM	DBE	Score	Formula
423.2071	423.2073	-0.2	-0.5	16.5	1	C28 H27 N2 O2
